# Action Recognition Depends on Observer’s Level of Action Control and Social Personality Traits

**DOI:** 10.1371/journal.pone.0081392

**Published:** 2013-11-22

**Authors:** Sasha Ondobaka, Roger D. Newman-Norlund, Floris P. de Lange, Harold Bekkering

**Affiliations:** 1 Donders Institute for Brain, Cognition and Behavior, Radboud University Nijmegen, Nijmegen, The Netherlands; 2 Department of Exercise Science, University of South Carolina, Columbia, South Carolina, United States of America; University G. d'Annunzio, Italy

## Abstract

Humans recognize both the movement (physical) goals and action (conceptual) goals of individuals with whom they are interacting. Here, we assessed whether spontaneous recognition of others’ goals depends on whether the observers control their own behavior at the movement or action level. We also examined the relationship between individual differences in empathy and ASD-like traits, and the processing of other individual’s movement and action goals that are known to be encoded in the “mirroring” and “mentalizing” brain networks. In order to address these questions, we used a computer-based card paradigm that made it possible to independently manipulate movement and action congruency of observed and executed actions. In separate blocks, participants were instructed to select either the right or left card (movement-control condition) or the higher or lower card (action-control condition), while we manipulated action- and movement-congruency of both actors’ goals. An action-congruency effect was present in all conditions and the size of this effect was significantly correlated with self-reported empathy and ASD-like traits. In contrast, movement-congruency effects were only present in the movement-control block and were strongly dependent on action-congruency. These results illustrate that spontaneous recognition of others’ behavior depends on the control scheme that is currently adopted by the observer. The findings suggest that deficits in action recognition are related to abnormal synthesis of perceived movements and prior conceptual knowledge that are associated with activations in the “mirroring” and “mentalizing” cortical networks.

## Introduction

The ability to recognize behavior of other agents is critical for successful social interaction, reproduction and survival [1]. Recognition of other individuals’ behavioral goals can occur either in a deliberate or a spontaneous manner [2], and at distinct levels of either physically defined movements or conceptually defined actions [3,4]. This ability to recognize others’ behavior is not equally present in all individuals. A profound disability in processing own and other individuals’ goals is associated with social and communicative challenges that are often observed in individuals diagnosed with autism spectrum disorder (ASD) [5-14]. 

The term ‘goal’ has often been used in a ill-defined manner within the field of action research [15,16]. A useful distinction in the alleged goal hierarchy [17-21] can be made between the level of *physical movement goals* which are associated with the kinematics of individual goal-directed movements (e.g., reaching towards the right), and *conceptual action goals*, which reflect functional expectations that govern movement execution (e.g., reaching to an object to drink coffee). Action goals include information about the expected external outcome of an action [22,23] and rely on prior conceptual knowledge [24] regarding relationships between objects in the environment and their purpose (e.g., cups are used to drink coffee). While physical movement goals typically involve a one-to-one perceptuo-motor mapping between the end-goal location and the observed movement kinematics [15,25-28], action goals may involve one-to-many mappings where a conceptual goal could be associated with expectation of multiple movement goals, dependent on the context in which behavior is embedded. In the present article, we use congruency effects between others’ and own behavior at the movement goal and at the action goal levels as an index for spontaneous goal recognition.

Neurally, the perceptuo-motor based account of action recognition suggests that the processing of other individuals’ goal-directed movements entails an automatic mapping to the observer’s own movement representations in the “action observation network/mirror neuron system (AON/MNS)” [6,7,29-31]. Prior behavioral research on action in social settings has mainly focused on movement goals, while neglecting the more complex conceptual action planning involved in everyday behavior [3]. In line with the perceptuo-motor account, it has repeatedly been shown that observation of another individual’s movement influences the observer’s subsequent execution of similar movements [32-36]. 

In contrast, in order to explain guidance of more complex social behavior, conceptual accounts of action understanding focus on the processing of other individuals’ conceptual action goals [37-39]. Cortical processing of action goals has been associated with activations in the “mentalizing/theory of mind network” comprising temporal and midline circuits, including medial parietal and prefrontal cortices and the superior temporal sulcus [40,41]. Recent behavioral work suggests that conceptual action goal congruency can have a powerful effect on subsequent action execution [4,42,43]. For example, we have recently suggested that during joint action observers rely on the same mechanism to process both their own action goals and the action goals of other individuals [4]. The results from this study also showed that when an observer’s behavioral control depends on the coactors goals (i.e., observer needed to match or mismatch coactors action goals), observed movement goals influence movement execution only when their action goals match. 

In contrast to the deliberate processing of others’ conceptual action goals [4], in many social situations individuals’ brains are spontaneously processing others’ behavior while simultaneously controlling their own action selection [2]. For example, spontaneous recognition of others’ behavioral goals appears in a situation in which you are having breakfast with your colleague. While you are reaching to the right for your knife, you might still be able to recognize your colleague’s movement goal towards a cup as well as the fact that she is having coffee; even though these goals are not directly related to the selection and control of your own behavior.

The current study had two primary aims. First, we assessed whether spontaneous goal recognition, as indexed by movement and action congruency effects, depends on the level of the control scheme adopted by the observer (i.e., movement or action). Second, to investigate a possible relationship between social personality traits and the level of social interaction, we asked whether these traits were related to congruency indices of action and movement goal recognition. We used a modified version of the card-selection paradigm employed by Ondobaka and colleagues (2012) to manipulate the congruency between participant’s and coactor’s conceptual action goals and physical movement goals. In the original experiment, participants needed to explicitly recognize coactor’s behavior in order to match or mismatch their action goal. In the current study, however, participants were explicitly instructed (written instructions prior to each block) to adopt specific types of control scheme independent of the coactor’s behavior. In 4 different blocks of trials, participants adopted a movement goal (i.e., movement-control condition) or an action goal (i.e., action-control condition) which guided the selection of one of two cards that were presented on a touch screen in front of them. In the movement-control blocks participants selected a card by focusing on its physical location (i.e., left or right), and in the action-control blocks they selected the card with the higher or lower value. During both the movement-control condition and the action-control condition participants observed the behavior of a confederate coactor who, immediately prior to the action of the participant, choose his own card. After the card-game, participants completed the Interpersonal Reactivity Index (IRI) [44] and the Autism-Spectrum Quotient (AQ) [45] that assessed their self-reported social personality traits. Whereas the IRI assesses individual differences in empathy on four subscales, the AQ provides a measure of the degree to which an individual with normal intelligence shows autistic traits. In short, the aim of this study was to test whether spontaneous recognition of other individuals’ behavioral goals is contingent on the behavioral control scheme adopted by the observer. Moreover, we were interested to examine whether individual differences in empathy and ASD personality traits would relate to movement goal congruency effects, or rather to conceptual action goal congruency effects. 

## Materials and Methods

### Participants and apparatus

**Figure 1:**
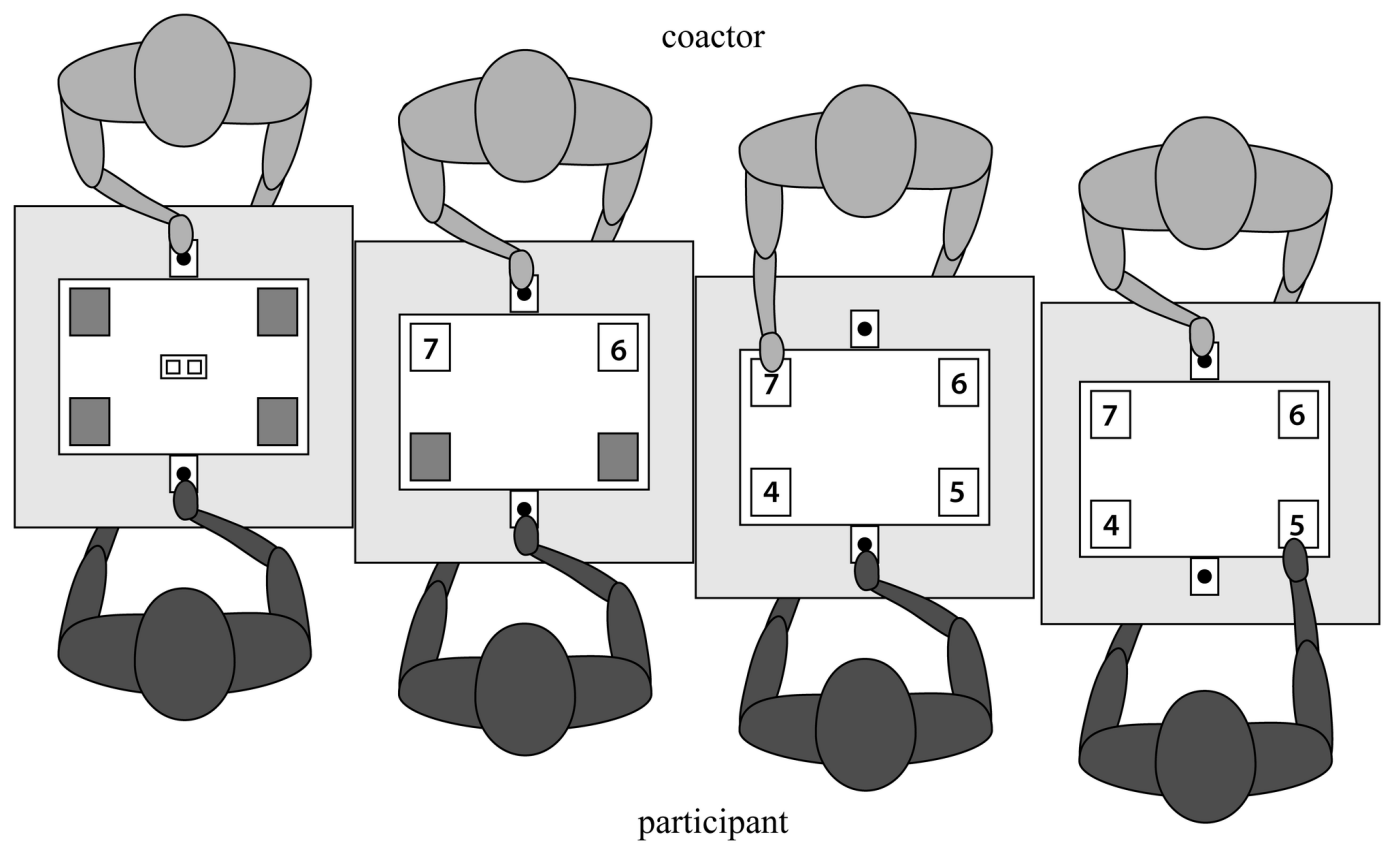
Experimental setup and illustration of a action/congruent-movement/congruent trial. Trials started with all four cards face down. After a variable delay (0.5–2.5 s), the two cards on the coactor's side were revealed. This triggered the coactor to make a selection, which caused the two cards on the participant's side to be revealed immediately. At this point, after having scanned all the revealed cards for the no-go cue (i.e., card with the value 2) the participant then made their choice according to the pertinent instruction for the given block. Immediately after selecting their cards by touching the screen, the coactor and participant placed their index fingers on their start buttons; the next trail was initiated when the coactor and then the participant had their fingers on the start buttons. The trial illustrated here is action congruent because both the coactor and the participant chose the card with the higher value; it is movement congruent because both the coactor and the participant moved to their right.

The study was approved by the Ethics Committee for Behavioural Research of the Social Sciences Faculty at Radboud University Nijmegen and is in accordance with the Helsinki declaration. All participants provided their written consent. Sixteen healthy (3 male), right-handed [46] participants were recruited from the Radboud University’s student population (mean age: 23,8 years; range: 21-31 years). Participants seated at a custom-built table (length = 120 cm, width = 80 cm) facing a male confederate coactor. A 19-in. touch screen (Elo Touch, Elo Touch Systems, Menlo Park, CA) and one start button on each long side of the screen were embedded in the table at a level even with the tabletop (see Figure **1** for an illustration of the experimental setup). Start buttons and the touch screen were connected to a PC using Presentation Software (Neurobehavioral systems Inc., Albany, CA). The program enabled us to detect stimulus onset, release of the start button (movement onset time) and contact with the touch screen (arrival time) with millisecond accuracy. Reaction time (RT) was calculated by subtracting the stimulus onset time from the movement onset time on each trial.

### Procedure, design, and stimuli

All participants performed the experimental task with the confederate coactor. On each trial, four cards were presented faced down, together with the start cue (Figure **1**). Next, the start cue disappeared and two cards were revealed to the confederate coactor, who had to select the card with either the higher or the lower value. So that the coactor would choose higher and lower cards at random, he was presented with either a high or low auditory cue at the beginning of each trial (i.e., the word *higher* or *lower*, audible only to the coactor through the earphones). The purpose of the start cue was solely to indicate the start of the trial. After a variable delay (0.5–2.5 s), the reminder cue disappeared, and the cards on the coactor's side of the screen were revealed. When the coactor chose one card by using his right index finger to press it on the touch screen, the cards on the participant's side of the screen were instantaneously revealed (see Figure 1). At this point, the participant used his or her right index finger to select a card according to the block instruction. Immediately after selecting their cards by touching the screen, the coactor and participant placed their index fingers on their start buttons. Finally, the next trail was initiated when the coactor and then the participant had their fingers on the start buttons. Participants were uninformed regarding whether the coactor had to select higher/lower card or left/right card. At the beginning of each block, the participant received an instruction to either select the left or the right card (i.e., movement-control condition) or the higher or the lower card (i.e., action-control condition) of the two cards presented in front of them. Additionally, both the participant and the coactor were instructed to adopt a predefined upright body posture, with the head in line with the centre of the touch screen and the start button. Furthermore, participants were explicitly instructed to constantly pay attention and be as fast and as accurate as possible throughout the experiment. 

To prevent the possible influence of ‘low level’ perceptual effects on RTs, the mapping of the cards was randomized in such a way that the 4 cards were always perceptually dissimilar. We took care that the lower/higher card appeared equally often on the left/right for both the coactor and the participant. Furthermore, the numerical distance between the value of the cards on both sides was always one. We also balanced the sum of the two cards such that it was equally likely to be higher or lower on any given trial. The card selected by the participant was independent of the card selected by the confederate coactor. However, in order to ensure that participants paid attention to the actions of the confederate coactor, they were required to scan all the revealed cards on the table and to refrain from making a response when a card with the value “2” (i.e., no-go cue) was revealed. This requirement was equal for all trials and did not bias participants’ performance towards any particular condition. No-go cards appeared in 23% of the trials in each block, and had an equal probability of appearing on the participant’s or the coactor’s side. At the level of movement goals, the responses of the coactor and the participant could be physically congruent (movement-congruent condition) or physically incongruent (movement-incongruent condition) from an egocentric perspective. Similarly, at the level of apparent action goals, the responses of the coactor and the participant could be action-congruent (both move to the high/low card) or action-incongruent (e.g., one moves to the high card while the other moves to the low card). 

We used similar 20 combinations of card values in all the conditions, while manipulating the spatial alignment of the target cards (i.e., whether the cards were presented at the same or different side from an egocentric perspective). The total of 104 trials (80 go and 24 no-go) from each of the 4 blocks (i.e., left, right, higher or lower control condition) were randomly drawn from this pool. The block instructions that manipulated movement-control (“Select the left/right card, unless a card with the value “2” appears on the screen”) or action-control (“Select the higher/lower card, unless a card with the value “2” appears on the screen) were presented on the screen for 10 s and were followed by the instruction to place the right index fingers at the start button to begin the block. Block order was counterbalanced between subjects using a pseudo-random sequence that resulted in 16 different combinations of the four blocks. We have excluded the no-go trials and trials in which participants or the coactor selected the incorrect card (1.0% of participants’ responses and 0.0% of the coactor’s responses) and in which response times (RTs) were more than 2.5 standard deviations above the mean (1.1% of participants’ responses and 1.2% of the coactor’s responses). After exclusion of participants´ and coactor´s responses that exceeded 2.5 SDs of each condition, mean response times were submitted to a 2 (control level: action or movement) x 2 (action-congruency: congruent or incongruent) x 2 (movement-congruency: congruent or incongruent) analysis of variance (ANOVA). After the participants completed the experiment, we asked them to fill in two self-report questionnaires. In order to assess the presence of individual empathic traits we used the *empathic concern* and the *perspective taking* subscales of the Interpersonal Reactivity Index [44]. To assess the individual ASD-like traits we used the Dutch version of the Autism-Spectrum Quotient [45].

## Results

**Figure 2:**
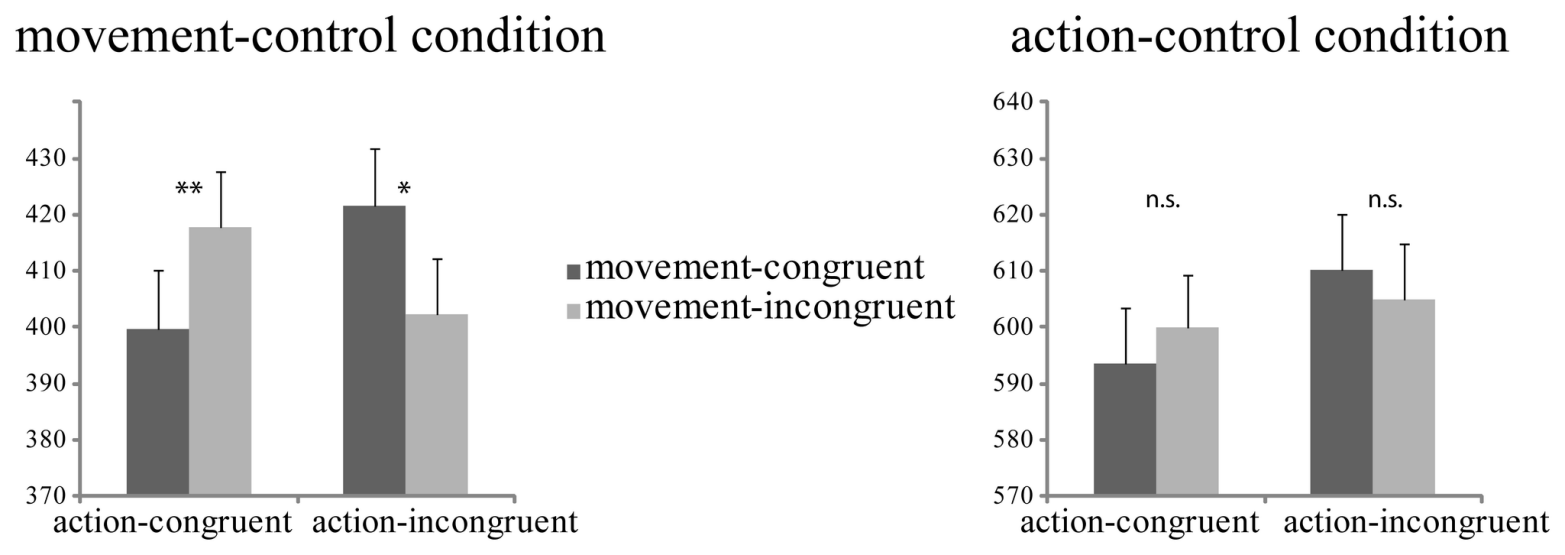
Participants’ mean response times in the action (conceptual)-control and movement (physical)-control conditions as a function of action (conceptual)-congruency and movement (physical)-congruency. Error bars represent standard errors of the mean (SEM). The asterisks indicate significant differences between conditions, * = *p* < .05, ** = *p* < .005.

Participants responded faster in the movement-control condition (mean RT = 410 ms, SEM = 21) compared to the action-control condition (mean RT = 602 ms, SEM = 18), *F*(1,15)= 224.55, *p* < .001; η^2^ = .94). Overall, we observed a significant action-congruency effect, participants were faster to select a card that had a relative value congruent to the coactor’s card value (mean RT = 503 ms, SEM = 19) as compared to the incongruent one (mean RT = 510 ms, SEM = 19), *F*(1,15)= 11.91, *p* = .004; η^2^ = .44. Mean response times in movement-congruent trials (mean RT = 506, SEM = 19) were not significantly different than those in the movement-incongruent trials (mean RT = 506, SEM = 19), *F*(1,15) = 0.00, *p* = .99; η^2^ = .00. A significant three-way interaction indicated that movement-congruency was dependent on action-congruency, solely in the movement-control condition (control level × action-congruency × movement-congruency, *F*(1,15) = 5.89, *p* = .03; η^2^ = .28, Figure **2**). To further examine the nature of the three-way interaction effect, we separately analyzed participants’ response times from the action-control and movement-control conditions.

### Response times in movement-control condition

In the movement-control condition, we found a significant two-way interaction between action-congruency and movement-congruency, *F* (1, 15) = 32.90, *p* < .001; η^2^ = .69 (see Figure **2**), indicating an interdependency between movement and action levels of processing. There was a marginally significant effect of action-congruency, *F* (1, 15) = 3.41, *p* = .09; η^2^ = .19; and no significant effect of movement-congruency, *F* (1, 15) = .02, *p* = .90; η^2^ = .00.

 To further specify the interaction effects in the movement-control condition, we ran post-hoc *t* tests to investigate movement-congruency effects separately for action-congruent and action-incongruent trials. When the chosen cards of the participant and coactor matched in relative value (i.e., their action goals were congruent), congruent movements were executed faster (mean RT = 400 ms, SEM = 21; see Figure **2**) than incongruent movements (mean RT = 418 ms, SEM = 21), *t* (1, 15) = 4.50, *p* < .001, d = -.22. When the relative value of the cards selected by the two actors was incongruent, incongruent movements were executed faster (mean RT = 402 ms, SEM = 22; see Figure **2**) than congruent movements (mean RT = 422 ms, SEM = 21), *t* (1, 15) = 2.53, *p* = .02, d = .23. In other words, when action goals matched, participants moved faster in the same direction as the confederate coactor, but when action goals clashed, movements in the opposite direction from the coactor were executed faster.

### Response times in action-control condition

In the action-control condition, participants responded faster in action-congruent trials (mean RT = 597 ms, SEM = 19 ms; see Figure **2**) than in the action-incongruent trials (mean RT = 608 ms, SEM = 18), *F* (1, 15) = 6.35, *p* = .02; η^2^ = .30. We observed neither significant movement-congruency effect, *F* (1, 15) = 0.02, *p* = .90; η^2^ = .00, nor an interaction between movement-congruency and action-congruency, *F* (1, 15) = 2.08, *p* = .17; η^2^ = .12.

### Accuracy scores

The participants showed high accuracy rates throughout the experiment. In the movement-control condition they made on average 0.15 % errors and in the action-control condition their average error rate was 0.11 %. The analysis of participants’ error rates revealed no significant results, all *p*’s > .1.

### Coactor’s response times

To examine whether the RTs of the coactor differed depending on the experimental conditions we ran a 2 (control level: action or movement) × 2 (action-congruency: congruent or incongruent) × 2 (movement-congruency: congruent or incongruent) ANOVA. As expected, the analysis showed no significant main effects, nor interaction effects (all ps > .1). These results indicate that the responses of the coactor were equally fast in all experimental conditions. We found no evidence that the RTs of the coactor influenced the RT effects of the participants.

### Correlation analysis between individual ASD-like and empathy trait scores and behavior

We ran a Spearman’s correlation analysis to examine whether individual differences in ASD-like traits and empathy were associated with the degree of individual action-congruency and movement-congruency effects. Individual congruency effects were obtained by calculating the overall differences between individual participants’ incongruent and congruent response times from action and movement congruency conditions. We used the acquired self-report scores from Autism-Spectrum Quotient [AQ, 45] and empathic concern (EC) and perspective taking (PT) subscales of Interpersonal Reactivity Index [IRI, 44] to assess individual personality differences in the tested pool of participants. EC scores [mean = 16.25 (SD = 3.49)] and PT [mean = 16.62 (SD = 4.99)] scores were comparable to the mean Dutch population scores [mean EC = 18.09 (SD = 4.23); mean PT = 17.29 (SD = 4.30)]. Similarly, participants’ AQ scores [mean = 13.50 (SD = 6.85)] were in line with the original control population scores [mean = 17.60 (SD = 6.40)]. As the mean score in the ASD population is 35.80 (SD = 6.50), a score of 32 on the AQ scale is suggested as a useful cutoff score for distinguishing between high-functioning individuals with ASD and controls [45]. All our participants had scores that were below 32 and were thus within the normal range.

**Figure 3:**
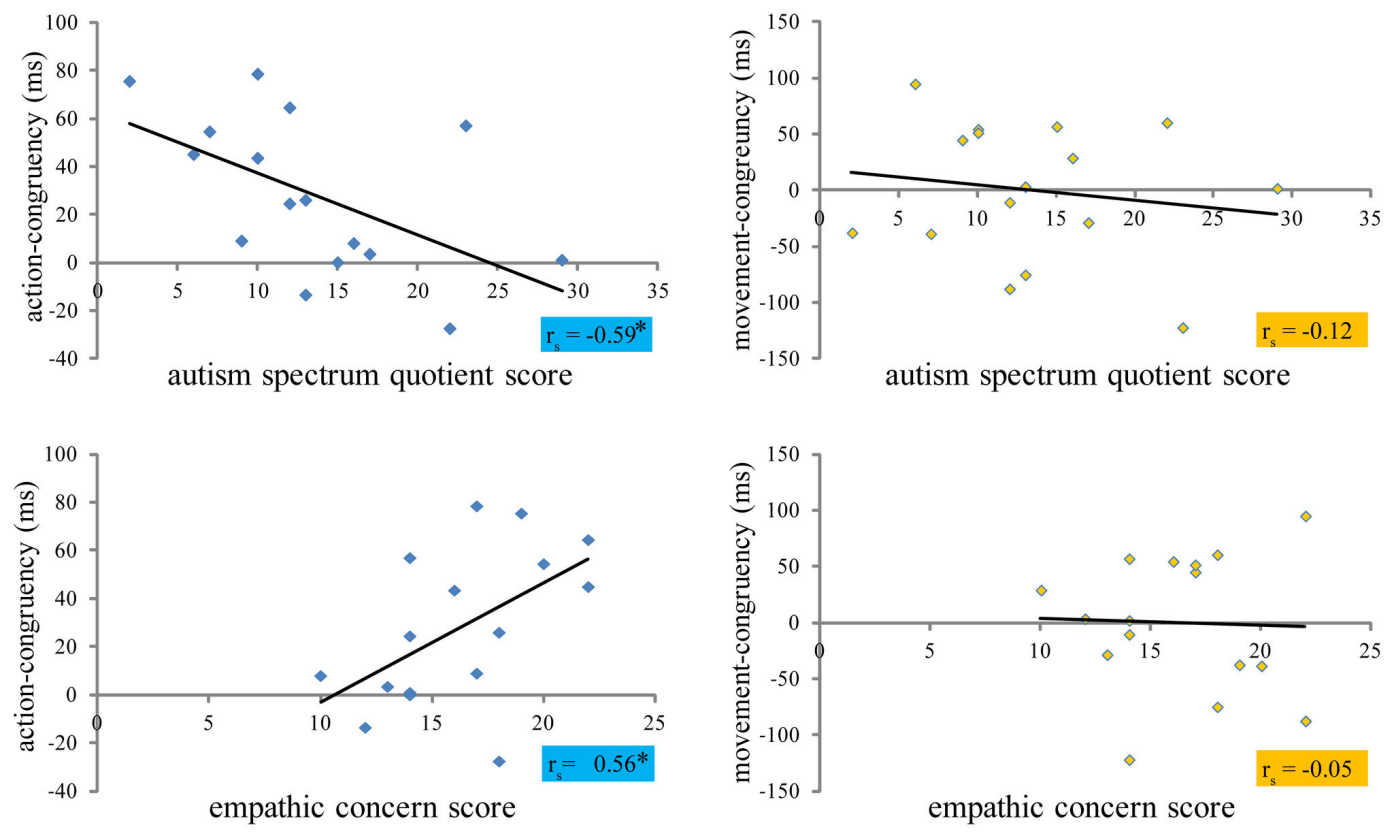
Participants’ individual action-congruency scores (left) and movement-congruency scores (right) as a function of their self-reported Autism spectrum quotient scores (AQ; top) and empathic concern scores (IRI; bottom); r values denote spearman’s rho correlation coefficients. Significant correlations, as indicated by the * were found for action-congruency scores, but not for movement-congruency scores, (*p* < .05).

Individual action-congruency effects were positively correlated with the empathy scores (*r*_*s*_ (16) = .55, *p* = .03) and perspective taking scores (*r*_*s*_ (16) = .41, *p* = .11) derived from the Interpersonal Reactivity Index (IRI), and negatively correlated with the Autism-Spectrum Quotient (AQ), *r*_*s*_ (16) = -.59, *p* = .02 (Figure **3**). That is, individuals that scored high on empathy and low on ASD-like traits were more strongly influenced by the conceptual action goals of the coactor. We did not observe any significant correlations between individual movement-congruency effects and either empathy scores, *r*_*s*_ (16) = .05, *p* = .87, or AQ scores, *r*_*s*_ (16) = -.12, *p* = .67. Similarly, no significant correlations were observed between the other two subscales of the IRI (fantasy and personal distress; all ps > .2) and either the movement- or action-congruency scores. In sum, the size of action-congruency effects, but not movement-congruency effects was related to the measured personality differences.

## Discussion

In social settings, humans constantly observe and expect behavior of other individuals in their surroundings. Here, we show that the spontaneous recognition of other individuals’ behavioral goals is contingent on the behavioral control scheme adopted by the observer during action observation. Specifically, when observers guided their behavior towards physical movement goals, their performance was affected by the expected conceptual action goals and the movement goals of the coactor. When observers guided their behavior based on action goals, their performance was only sensitive to the coactors action goals. These findings are informative regarding the functional action hierarchy that underlies action and movement control of behavior in social interaction. Moreover, we report a relationship between individual differences in the size of action congruency effects and individual differences in empathy and ASD personality traits, while no relationship was found for movement congruency effects. 

### Shared hierarchy for action recognition and action control

There is a plethora of evidence that supports hierarchical models of neurocognitive processing, but a general characterization of the different components within each distinct functional and neural hierarchy is still missing [16,21,47-50]. Our novel finding, namely, that how observers control their behavior alters the influence of observed behavior on their movements, is consistent with the idea that the multitiered system underlies both action control and action recognition. The data is in line with and builds upon previous work demonstrating that, in situations in which observer’s behavior is directly related to others’ behavior, performance is altered by congruency between observer’s and coactor’s movement goals e.g. [33,35] and action goals [4,43]. Our findings indicate that action goal congruency between the coactors plays an important role in social interaction, irrespective of the level of behavioral control. Movement goal congruency appeared to be only relevant in cases where the observer controlled their behavior at the movement level.

One interesting finding to emerge from the present study is that a mismatch between observers’ and coactors’ action goals resulted in slower response times for congruent movement goals. This reversal of the movement-congruency effect is in line with the recent findings [28,51], which showed that one-to-one associations between observed and executed action can be modulated by the context in which the actions are embedded. Our findings extend previous work by demonstrating that a spontaneous recognition of other individual’s potential action goals (e.g., choosing a higher card of the two) [22,23,52] affects observer’s movement execution and alters the influence of other’s movements on the observer’s movement execution [15,25-28]. One could argue that participants ‘automatically’ took into account the mapping of the numbers, irrespective of movement- or action-congruency. However, we assert that merely visual mapping of the cards cannot explain the reported findings. Spatial mapping of the cards should be regarded as a necessary, but not sufficient, contextual condition for adaptive perceptuo-motor coupling during both action control and action recognition. The interpretation rather requires the assumption that participants have conceptual (relational) model (which of the two cards is higher) of the objects in the environment, which dynamically modulates the perceptuo-motor mapping that underlies the body-world interaction [53]. 

A probabilistic conceptuo-perceptual framework [53] for action control and action recognition offers a possible explanation for the reversal of the movement-congruency effects. This framework assumes that both control and recognition of action depend on combining prior conceptual information about the relationships between the objects and the immediate perceptuo-motor information regarding movement selection. In the present experiment, incongruent conceptual expectations (i.e. mismatch between the conceptual goals of the actor and coactor) may have facilitated the selection of a movement direction incongruent to that of the coactor, or, alternatively inhibited the selection of a congruent movement. Similar, but opposite facilitatory and inhibitory effects would be predicted in the case that the actor’s and coactor’s conceptual expectations matched (i.e., both going for the low card, or both going for the high card). The conceptuo-perceptual probabilistic framework described here can contribute to a better understanding of the mechanisms that underlie flexible selection and recognition of movements in social situations [28,53,54].

A lot of controversy exists regarding the exact neural instantiation of action recognition [29,37,38,55-58]. Our behavioral findings point to the notion that a dynamic interplay between mentalizing and AON/MNS brain networks might underpin spontaneous recognition of observed everyday behavior [21,41,59,60]. The results of the current study are in line with dynamic multitiered accounts of action representation [17,19,21,61,62] and are consistent with the idea that processing behavior can be viewed as the synthesis of immediate movement information and prior conceptual knowledge. The formalization of appropriate multitiered models and illuminating their neural correlates is critical to understanding guidance of everyday social behaviors [3,21,52,63-65]. 

### Relation between personality traits and hierarchical goal processing

The neurocognitive basis of social impairments in ASD is currently heavily debated in cognitive neurosciences [31,66,67]. Both perceptuo-motor theories [6,7,12,31] and conceptual theories [9,11,39,68,69] have been proposed to explain the social and communicative deficits typically observed in the ASD population. Here, we showed that self-reported autistic characteristics and empathy are related to spontaneous recognition of conceptual action goals, rather than physical movement goals. Studies on the association between the ability to process human movements and ASD have reported mixed results [66,67]. Some researchers found evidence for impaired processing of physical movement goals in ASD [6,7,13,14], whereas others were unable to find such an association, e.g. [ 70,71]. 

We show a relationship between conditions characterized by social deficits and the ability to process conceptual action goals, which are distinct from the corresponding physical movements [13,25,70,71], but pertain to overarching prior knowledge driving purposeful action [23,52,63-65]. Synthesis of conceptual knowledge that is associated with brain activity in the ‘mentalizing’ regions [72] and the observed movement goals, processed by the parieto-frontal ‘mirroring’ circuit [29,31] is necessary for the inference of others’ object-related conceptual action goals [3,21,41,65,73]. Nevertheless, given the intertwined nature of conceptual and perceptuo-motor representations, the lack of correlation between movement goal recognition and ASD characteristics should not be taken as direct evidence against the perceptuo-motor explanations for impairments in action inference. Rather, the findings raise the interesting possibility that ASD symptoms result from dysfunctional spontaneous integration of observed goal-directed movements with the observer’s contextually activated conceptual knowledge, which might entail the interaction between the cortical networks for “mentalizing” and “action observation” [2,21,41,59,60]. Present results suggest that social learning at a conceptual level, or rather the interaction between conceptual level and observed movements, might be impaired in the ASD population. Adopting the conceptuo-movement hierarchical approach of action processing [53] could lead to novel insights regarding such aberrant social interaction. For example, a key tenet of the conceptuo-perceptual approach, that processing of behavior entails a synthesis of immediate movement information and prior conceptual knowledge, could guide development of new forms of therapy for ASD individuals.

## Conclusion

In sum, our findings are consistent with the idea that spontaneous recognition of others’ behavioral goals depends on the observer’s level of behavioral control. We suggest that empathy and ASD-like personality characteristics might be related to an impairment in the synthesis of incoming perceptuo-motor information and prior conceptual knowledge related to functional outcomes in the world. The results also indicate a need to further investigate the roles of parieto-frontal “action observation” and medial-temporal “mentalizing” brain networks that are known for their involvement in processing movement and conceptual information.

## References

[1] Tomasello M (1999) The human adaptation for culture. Annual Review of Anthropology: 509-529.

[2] Adolphs R (2009) The social brain: neural basis of social knowledge. Annu Rev Psychol 60: 693-716. doi:10.1146/annurev.psych.60.110707.163514. PubMed: 18771388. 18771388 PMC2588649

[3] Ondobaka S, Bekkering H (2012) Hierarchy of idea-guided action and perception-guided movement. Frontiers in Cognition 3: 1-5. 10.3389/fpsyg.2012.00579 PMC3573717 23423700

[4] Ondobaka S, De Lange FP, Newman-Norlund RD, Wiemers M, Bekkering H (2012) Interplay between action and movement intentions during social interaction. Psychol Sci 23: 30-35. doi:10.1177/0956797611424163. PubMed: 22157675. 22157675

[5] Senju A, Southgate V, Miura Y, Matsui T, Hasegawa T et al. (2010) Absence of spontaneous action anticipation by false belief attribution in children with autism spectrum disorder. Dev Psychopathol 22: 353-360. doi:10.1017/S0954579410000106. PubMed: 20423546. 20423546

[6] Cattaneo L, Fabbri-Destro M, Boria S, Pieraccini C, Monti A et al. (2007) Impairment of actions chains in autism and its possible role in intention understanding. Proc Natl Acad Sci U S A 104: 17825-17830. doi:10.1073/pnas.0706273104. PubMed: 17965234. 17965234 PMC2077067

[7] Dapretto M, Davies MS, Pfeifer JH, Scott AA, Sigman M et al. (2006) Understanding emotions in others: mirror neuron dysfunction in children with autism spectrum disorders. Nat Neurosci 9: 28-30. PubMed: 16327784. 16327784 10.1038/nn1611 PMC3713227

[8] Haswell CC, Izawa J, Dowell LR, Mostofsky SH, Shadmehr R (2009) Representation of internal models of action in the autistic brain. Nat Neurosci 12: 970-972. doi:10.1038/nn.2356. PubMed: 19578379. 19578379 PMC2740616

[9] Baron-Cohen S, Leslie AM, Frith U (1985) Does the autistic child have a theory of mind? Cognition 21: 37-46. doi:10.1016/0010-0277(85)90022-8. PubMed: 2934210. 2934210

[10] Frith U, Morton J, Leslie AM (1991) The cognitive basis of a biological disorder. Autism - Trends in Neurosciences 14: 433-438. doi:10.1016/0166-2236(91)90041-R. 1722361

[11] Leslie AM, Thaiss L (1992) Domain specificity in conceptual development: Neuropsychological evidence from autism. Cognition 43: 225-251. doi:10.1016/0010-0277(92)90013-8. PubMed: 1643814. 1643814

[12] Williams JH, Waiter GD, Gilchrist A, Perrett DI, Murray AD et al. (2006) Neural mechanisms of imitation and 'mirror neuron' functioning in autistic spectrum disorder. Neuropsychologia 44: 610-621. doi:10.1016/j.neuropsychologia.2005.06.010. PubMed: 16140346. 16140346

[13] Fabbri-Destro M, Cattaneo L, Boria S, Rizzolatti G (2009) Planning actions in autism. Exp Brain Res 192: 521-525. doi:10.1007/s00221-008-1578-3. PubMed: 18839160. 18839160

[14] Theoret H, Halligan E, Kobayashi M, Fregni F, Tager-Flusberg H et al. (2005) Impaired motor facilitation during action observation in individuals with autism spectrum disorder. Current Biology 15: R84-R85. doi:10.1016/j.cub.2005.01.022. PubMed: 15694294. Available online at: 10.1016/j.cub.2005.01.022 Available online at: PubMed: 15694294 15694294

[15] Leighton J, Bird G, Heyes C (2010) 'Goals' are not an integral component of imitation. Cognition 114: 423-435. doi:10.1016/j.cognition.2009.11.001. PubMed: 19945096. 19945096

[16] Uithol S, van Rooij I, Bekkering H, Haselager P (2012) Hierarchies in Action and Motor Control. J Cogn Neurosci 24: 1077-1086. doi:10.1162/jocn_a_00204. PubMed: 22288396. 22288396

[17] Pacherie E (2008) The phenomenology of action: A conceptual framework. Cognition 107: 179-217. doi:10.1016/j.cognition.2007.09.003. PubMed: 17950720. 17950720

[18] Searle JR (1983) Intentionality, an essay in the philosophy of mind. New York, NY: Cambridge University Press.

[19] Vallacher RR, Wegner DM (1987) What do people think they're doing? Action identification and human behavior. Psychological Review 94: 3-15. doi:10.1037/0033-295X.94.1.3.

[20] Grafton ST, Hamilton CAF (2007) Evidence for a distributed hierarchy of action representation in the brain. Hum Mov Sci 26: 590-616. doi:10.1016/j.humov.2007.05.009. PubMed: 17706312. 17706312 PMC2042582

[21] Kilner JM (2011) More than one pathway to action understanding. Trends Cogn Sci 15: 352-357. doi:10.1016/j.tics.2011.06.005. PubMed: 21775191. 21775191 PMC3389781

[22] Hodges JR, Bozeat S, Ralph MAL, Patterson K, Spatt J (2000) The role of conceptual knowledge in object use evidence from semantic dementia. Brain 123: 1913-1925. doi:10.1093/brain/123.9.1913. PubMed: 10960055. 10960055

[23] Nakayama Y, Yamagata T, Tanji J, Hoshi E (2008) Transformation of a virtual action plan into a motor plan in the premotor cortex. J Neurosci 28: 10287-10297. doi:10.1523/JNEUROSCI.2372-08.2008. PubMed: 18842888. 18842888 PMC6671018

[24] Peelen MV, Caramazza A (2012) Conceptual object representations in human anterior temporal cortex. J Neurosci 32: 15728-15736. doi:10.1523/JNEUROSCI.1953-12.2012. PubMed: 23136412. 23136412 PMC6621609

[25] Bekkering H, Wohlschläger A, Gattis M (2000) Imitation of gestures in children is goal-directed. Quarterly Journal of Experimental Psychology: Section A 53: 153-164. doi:10.1080/713755872. PubMed: 10718068. 10718068

[26] Iacoboni M, Woods RP, Brass M, Bekkering H, Mazziotta JC et al. (1999) Cortical mechanisms of human imitation. Science 286: 2526–2528. doi:10.1126/science.286.5449.2526. PubMed: 10617472. 10617472

[27] Liepelt R, Von Cramon DY, Brass M (2008) What is matched in direct matching? Intention attribution modulates motor priming. Journal of Experimental Psychology 34: 578-591. PubMed: 18505325. 18505325 10.1037/0096-1523.34.3.578

[28] Van Schie HT, van Waterschoot BM, Bekkering H (2008) Understanding action beyond imitation: Reversed compatibility effects of action observation in imitation and joint action. J Exp Psychol Hum Percept Perform 34: 1493-1500. doi:10.1037/a0011750. PubMed: 19045988. 19045988

[29] Rizzolatti G, Sinigaglia C (2010) The functional role of the parieto-frontal mirror circuit: interpretations and misinterpretations. Nat Rev Neurosci 11: 264-274. doi:10.1038/nrn2805. PubMed: 20216547. 20216547

[30] Heyes C (2011) Automatic imitation. Psychol Bull 137: 463-483. doi:10.1037/a0022288. PubMed: 21280938. 21280938

[31] Rizzolatti G, Fabbri-Destro M, Cattaneo L (2009) Mirror neurons and their clinical relevance. Nat Clin Pract Neurol 5: 24-34. doi:10.1038/ncpendmet1007. PubMed: 19129788. 19129788

[32] Brass M, Bekkering H, Prinz W (2001) Movement observation affects movement execution in a simple response task. Acta Psychol (Amst) 106: 3-22. doi:10.1016/S0001-6918(00)00024-X. PubMed: 11256338. 11256338

[33] Brass M, Bekkering H, Wohlschläger A, Prinz W (2000) Compatibility between Observed and Executed Finger Movements: Comparing Symbolic, Spatial, and Imitative Cues. Brain Cogn 44: 124-143. doi:10.1006/brcg.2000.1225. PubMed: 11041986. 11041986

[34] Chartrand TL, Bargh JA (1999) The chameleon effect: The perception–behavior link and social interaction. J Pers Soc Psychol 76: 893-910. doi:10.1037/0022-3514.76.6.893. PubMed: 10402679. 10402679

[35] Kilner JM, Paulignan Y, Blakemore SJ (2003) An interference effect of observed biological movement on action. Curr Biol 13: 522-525. doi:10.1016/S0960-9822(03)00165-9. PubMed: 12646137. 12646137

[36] Heyes C, Bird G, Johnson H, Haggard P (2005) Experience modulates automatic imitation. Brain Res Cogn Brain Res 22: 233-240. doi:10.1016/j.cogbrainres.2004.09.009. PubMed: 15653296. 15653296

[37] Hauser M, Wood J (2010) Evolving the capacity to understand actions, intentions, and goals. Annu Rev Psychol 61: 303-324. doi:10.1146/annurev.psych.093008.100434. PubMed: 19575605. 19575605

[38] Csibra G (2008) Action mirroring and action understanding: An alternative account. In: Haggard P Rosseti Y Kawato M Sensorimotor foundations of higher cognition. Oxford: Oxford University Press pp. 435-480.

[39] Frith CD, Frith U (1999) Interacting minds--a biological basis. Science 286: 1692-1695. doi:10.1126/science.286.5445.1692. PubMed: 10576727. 10576727

[40] Brass M, Schmitt RM, Spengler S, Gergely G (2007) Investigating action understanding: Inferential processes versus action simulation. Curr Biol 17: 2117-2121. doi:10.1016/j.cub.2007.11.057. PubMed: 18083518. 18083518

[41] De Lange FP, Spronk M, Willems RM, Toni I, Bekkering H (2008) Complementary systems for understanding action intentions. Curr Biol 18: 454-457. doi:10.1016/j.cub.2008.02.057. PubMed: 18356050. 18356050

[42] Massen C, Prinz W (2007) Activation of action rules in action observation. J Exp Psychol Learn Mem Cogn 33: 1118-1130. doi:10.1037/0278-7393.33.6.1118. PubMed: 17983317. 17983317

[43] Massen C, Prinz W (2009) Movements, actions and tool-use actions: an ideomotor approach to imitation. Philos Trans R Soc Lond B Biol Sci 364: 2349-2358. doi:10.1098/rstb.2009.0059. PubMed: 19620106. 19620106 PMC2865071

[44] Davis MH (1983) Measuring individual differences in empathy: Evidence for a multidimensional approach. Journal of Personality and Social Psychology 44: 113-126. doi:10.1037/0022-3514.44.1.113.

[45] Baron-Cohen S, Wheelwright S, Skinner R, Martin J, Clubley E (2001) The Autism-Spectrum Quotient (AQ): Evidence from Asperger Syndrome/High-Functioning Autism, Males and Females, Scientists and Mathematicians. Journal of Autism and Developmental Disorders 31: 5-17. doi:10.1023/A:1005653411471. PubMed: 11439754. 11439754

[46] Oldfield RC (1971) The assessment and analysis of handedness: the Edinburgh inventory. Neuropsychologia 9: 97-113. doi:10.1016/0028-3932(71)90067-4. PubMed: 5146491. 5146491

[47] Badre D, D'Esposito M (2009) Is the rostro-caudal axis of the frontal lobe hierarchical? Nat Rev Neurosci 10: 659-669. doi:10.1038/nrg2679. PubMed: 19672274. 19672274 PMC3258028

[48] Hamilton AFC, Grafton ST (2007) The motor hierarchy: from kinematics to goals and intentions. Sensorimotor Foundations of Higher Cognition 22: 381-408.

[49] Botvinick MM (2008) Hierarchical models of behavior and prefrontal function. Trends Cogn Sci 12: 201-208. doi:10.1016/j.tics.2008.02.009. PubMed: 18420448. 18420448 PMC2957875

[50] Fuster JM (2004) Upper processing stages of the perception-action cycle. Trends Cogn Sci 8: 143-145. doi:10.1016/j.tics.2004.02.004. PubMed: 15551481. 15551481

[51] Cook R, Dickinson A, Heyes C (2012) Contextual Modulation of Mirror and Countermirror Sensorimotor Associations. J Exp Psychol Gen 141: 774-787. doi:10.1037/a0027561. PubMed: 22428612. 22428612

[52] Tanji J, Shima K, Mushiake H (2007) Concept-based behavioral planning and the lateral prefrontal cortex. Trends Cogn Sci 11: 528-534. doi:10.1016/j.tics.2007.09.007. PubMed: 18024183. 18024183

[53] Ondobaka S, Bekkering H (. (2013)) Conceptual and perceptuo-motor action control and action recognition. Cortex. PubMed: 23932742 10.1016/j.cortex.2013.06.005 23932742

[54] Newman-Norlund RD, van Schie HT, van Zuijlen AMJ, Bekkering H (2007) The mirror neuron system is more active during complementary compared with imitative action. Nat Neurosci 10: 817-818. doi:10.1038/nn1911. PubMed: 17529986. 17529986

[55] Jacob P, Jeannerod M (2005) The motor theory of social cognition: a critique. Trends Cogn Sci 9: 21-25. doi:10.1016/j.tics.2004.11.003. PubMed: 15639437. 15639437

[56] Hickok G (2009) Eight problems for the mirror neuron theory of action understanding in monkeys and humans. J Cogn Neurosci 21: 1229-1243. doi:10.1162/jocn.2009.21189. PubMed: 19199415. 19199415 PMC2773693

[57] Bekkering H, De Bruijn ERA, Cuijpers RH, Newman Norlund R, Van Schie HT et al. (2009) Joint action: Neurocognitive mechanisms supporting human interaction. Topics Cognitive Science 1: 340-352. doi:10.1111/j.1756-8765.2009.01023.x. 25164937

[58] Ferrari PF, Bonini L, Fogassi L (2009) From monkey mirror neurons to primate behaviours: possible 'direct' and 'indirect' pathways. Philos Trans R Soc Lond B Biol Sci 364: 2311-2323. doi:10.1098/rstb.2009.0062. PubMed: 19620103. 19620103 PMC2865083

[59] Keysers C, Gazzola V (2007) Integrating simulation and theory of mind: from self to social cognition. Trends Cogn Sci 11: 194-196. doi:10.1016/j.tics.2007.02.002. PubMed: 17344090. 17344090

[60] Uddin LQ, Iacoboni M, Lange C, Keenan JP (2007) The self and social cognition: the role of cortical midline structures and mirror neurons. Trends Cogn Sci 11: 153-157. doi:10.1016/j.tics.2007.01.001. PubMed: 17300981. 17300981

[61] Waytz A, Gray K, Epley N, Wegner DM (2010) Causes and consequences of mind perception. Trends Cogn Sci 14: 383-388. doi:10.1016/j.tics.2010.05.006. PubMed: 20579932. 20579932

[62] Kilner JM, Friston KJ, Frith CD (2007) Predictive coding: an account of the mirror neuron system. Cogn Process 8: 159-166. doi:10.1007/s10339-007-0170-2. PubMed: 17429704. 17429704 PMC2649419

[63] Hodges JR, Spatt J, Patterson K (1999) "What" and "how": evidence for the dissociation of object knowledge and mechanical problem-solving skills in the human brain. Proceedings of the National Academy of Sciences of the USA 96: 9444-9448. doi:10.1073/pnas.96.16.9444. 10430962 PMC17802

[64] Roy EA, Square PA (1985) Common considerations in the study of limb, verbal and oral apraxia. Advances in Psychology 23: 111-161. doi:10.1016/S0166-4115(08)61139-5.

[65] Johnson-Frey SH (2003) What's so special about human tool use? Neuron 39: 201-204. doi:10.1016/S0896-6273(03)00424-0. PubMed: 12873378. 12873378

[66] Hamilton AFC (2009) Research review: Goals, intentions and mental states: Challenges for theories of autism. Journal of Child Psychology and Psychiatry 50: 881-892. doi:10.1111/j.1469-7610.2009.02098.x. PubMed: 19508497. 19508497

[67] Hamilton AFC (2013) Reflecting on the mirror neuron system in autism: A systematic review of current theories. Developmental. Journal of Cognitive Neuroscience 3: 91-105. doi:10.1016/j.dcn.2012.09.008. PMC6987721 23245224

[68] Moran JM, Young LL, Saxe R, Lee SM, O'Young D et al. (2011) Impaired theory of mind for moral judgment in high-functioning autism. Proc Natl Acad Sci U S A 108: 2688-2692. doi:10.1073/pnas.1011734108. PubMed: 21282628. 21282628 PMC3041087

[69] Senju A, Southgate V, White S, Frith U (2009) Mindblind eyes: an absence of spontaneous theory of mind in Asperger syndrome. Science 325: 883-885. doi:10.1126/science.1176170. PubMed: 19608858. 19608858

[70] Bird G, Leighton J, Press C, Heyes C, Bird G et al. (2007) Intact automatic imitation of human and robot actions in autism spectrum disorders. Proceedings of the Royal Society of London B. Biological Sciences 274: 3027-3031. doi:10.1098/rspb.2007.1019. PubMed: 17911053. PMC2291158 17911053

[71] Avikainen S, Wohlschläger A, Liuhanen S, Hänninen R, Hari R (2003) Impaired mirror-image imitation in Asperger and high-functioning autistic subjects. Curr Biol 13: 339-341. doi:10.1016/S0960-9822(03)00265-3. PubMed: 12593801. 12593801

[72] Frith CD, Frith U (2006) The neural basis of mentalizing. Neuron 50: 531-534. doi:10.1016/j.neuron.2006.05.001. PubMed: 16701204. 16701204

[73] Wang Y, Hamilton AFC (2012) Social top-down response modulation (STORM): a model of the control of mimicry in social interaction. Frontiers in Human Neuroscience 6: 1-10. PubMed: 22279433. 22675295 10.3389/fnhum.2012.00153 PMC3366585

